# Use of Counterfactual Population Projections for Assessing the Demographic Determinants of Population Ageing

**DOI:** 10.1007/s10680-020-09567-9

**Published:** 2020-09-09

**Authors:** Michael Murphy

**Affiliations:** London School of Economics and Political Science, London, UK

**Keywords:** Demography, Population projections, Population ageing, Long-term trends

## Abstract

Counterfactual population projections have been used to estimate the contributions of fertility and mortality to population ageing, a method recently designated as the gold standard for this purpose. We analyse projections with base years between 1850 and 1950 for 11 European countries with long-run demographic data series to estimate the robustness of this approach. We link this approach with stable population theory to derive quantitative indicators of the role of fertility and mortality; consider ways of incorporating net migration; and examine the effect of using alternative indicators of population ageing. A number of substantive and technical weaknesses in the counterfactual projection approach are identified: (1) the conclusions are very sensitive to the choice of base year. Specifically, the level of base year fertility has a major influence on whether fertility or mortality is considered the main driver of population ageing. (2) The method is not transitive: results for two adjacent intervals are unrelated to results for the combined period. Therefore, overall results cannot be usefully allocated between different sub-intervals. (3) Different ageing indices tend to produce similar qualitative conclusions, but quantitative results may differ markedly. (4) Comparisons of alternative models should be with a fixed fertility and mortality projection model rather than with the baseline values as usually done. (5) The standard counterfactual projections approach concatenates the effects of initial age structure and subsequent fertility and mortality rates: methods to separate these components are derived.

## Introduction

The latest United Nations population projections suggest that by 2100 in Europe, one person in seven will be aged 80 or over and over 30 per cent of people in three quarters of European countries will be aged 65 or over (United Nations Department of Economic and Social Affairs 2019). Population ageing is of increasing importance for both demographic and policy planning. The Director of the UN Population Division, John Wilmoth, stated in 2015: “For many countries today, and probably for most countries in the long run, the major concern about their demographic situation will be in relation to population ageing, not growth” (Wilmoth 2015a).

Population size and structure are determined by natural change—births minus deaths—and net migration. Mortality improvement has sometimes been presented as the plausible primary driver of long-term population ageing trends since it is the driver of individual ageing. However, primacy has usually been given to fertility decline ever since the determinants of population ageing became a topic of study from the 1950s (e.g. Coale 1956; United Nations 1956; Valaoras 1950, 1958). While mortality improvement is recognised as an increasingly important factor in population ageing, especially in below replacement-level fertility societies where the majority of the world’s population now live, the standard view remains that “fertility decline is, by far, the most important cause of population ageing” (Wilmoth 2015b). The long-term effect of migration on population ageing is generally regarded as minor in most situations (Goldstein 2009; Murphy 2016a).

Population ageing is closely linked to demographic transition, although it occurs with a considerable lag compared with mortality and fertility change. In the pre-transition phase, fertility and mortality are broadly constant, and so populations do not age. When fertility declines, populations start to age, but this is initially offset by mortality improvement being particularly concentrated at younger ages, which has the effect of making population structures younger rather than older. At a later stage, mortality improvement occurs mainly at older ages and reinforces population ageing, becoming increasingly important when life expectancy at birth increases beyond about 70 years, which occurred around the 1950s in Europe (Lee 1994). In the extended post-transition period in high-income countries when fertility had been relatively constant, mortality improvement concentrated at older ages comes to dominate population ageing.

Conclusions that fertility had a dominant role have usually been based on two approaches: (1) static stable population models such as those of Coale (1956) and (2) studies comparing actual out-turns with counterfactual population projections, usually with constant fertility or mortality rates from an earlier baseline (e.g. Hermalin 1966; Notestein 1960; Valaoras 1950). Bengtsson and Scott (2010, 2011), assuming constant fertility in Sweden over the twentieth century, concluded that declining fertility was the primary cause of population ageing and that declining mortality started to become influential only towards the end of the twentieth century. Lee and Zhou (2017) applied counterfactual population projections to Indian data with base year of 1900 and More Developed Countries (MDCs) from 1910. They also concluded that fertility decline had been the main reason for population ageing in both areas and they argued that this method is the “gold standard” for assessing the relative contributions of fertility and mortality to population ageing (Population and Development Review 2017, p. 394).

Preston et al. (1989) developed a method based on the Preston-Coale synthesis (Preston and Coale 1982) that decomposes actual changes in population mean age between births, mortality and net migration. They concluded that mortality improvement was the main driver of population ageing in two developed countries, USA and Sweden, in the period 1985–1990. Preston and Stokes (2012) used the same model and argued that improvement in mortality in successive birth cohorts was the most important source of population ageing, accounting for 82 per cent in the period 2005–2010 in high-income countries (excluding Eastern Europe), and that in middle- and low-income countries, the contributions of mortality and fertility declines to population ageing were similar (Preston and Stokes 2012, Table 1). Recently, Murphy (2017) has extended the Preston, Himes and Eggers model to incorporate fertility rates into the model and obtained estimates of the relative contribution of fertility, mortality and net migration to population ageing for 11 European countries across the whole of the twentieth century. These results confirm that the importance of mortality decline on population ageing holds over an extended set of countries and time frames.

**Table 1:** Projection Scenarios

Scenario	Description	Rates		Age structure index at time *t*	Age structure index determined by
		Fertility	Mortality		
*Ac*	Actual	Actual changes	Actual changes	*a*^Ac^(*t*)	Initial population; subsequent actual net migration, fertility and mortality
*Bf*	Both fixed	Fixed at initial rates	Fixed at initial rates	*a*^Bf^(*t*)	Initial population, fertility and mortality; subsequent actual net migration
Mf	Mortality fixed	Actual changes	Fixed at initial rates	a^Mf^(t)	Initial population and mortality; subsequent actual net migration and fertility
Ff	Fertility fixed	Fixed at initial rates	Actual changes	a^Ff^(t)	Initial population and fertility; subsequent actual net migration and mortality

Age structure does not depend on migration in zero migration scenarios

Most studies using counterfactual population projections have concluded that fertility change has been the primary cause of population ageing. However, to our knowledge, there is no comprehensive and systematic investigation of the robustness and sensitivity of this approach for analysing the determinants of population ageing. In this paper, we compare alternative projection approaches over different time periods for a number of high-income countries. The paper is organised as follows: we produce sets of population projections for European countries with consistent data from the nineteenth century for different base years with constant fertility and mortality in order to:

1. Assess the sensitivity of conclusions about the determinants of population ageing to the choice of alternative projection bases.
2. Develop and present methods to quantify the relative contributions of fertility and mortality to population ageing.
3. Provide detailed analyses for three countries on the effects of including or excluding migration.
4. Consider the use of different indicators for analysing population ageing.

Finally, we draw general conclusions about the strengths and weaknesses of counterfactual population projections for elucidating the determinants of population ageing.

## Data and methods

Data are taken from the Human Mortality Database (HMD), which contains estimated mortality rates and population sizes by single year of age and sex for each calendar year from around the time of the start of national vital registration. These estimates are based on information from official statistics such as censuses, vital registration, and population estimates (Human Mortality Database 2018; Wilmoth et al. 2007). The database also includes information on total annual number of births and deaths. Mortality rates are available in both period and cohort form for most years. Since our principal interest is in comparing national values across extended time scales, we use 11 countries that have data extending back to the nineteenth century, concentrating on three of these countries with different trajectories, England and Wales from 1841, France from 1815 and Sweden from 1751. Data are available up to around 2015.

We use a standard cohort component projection model. Cohort mortality rates are not available in HMD for those born after the early 1980s, so we use period mortality rates to estimate survival for these cohorts. Annual information on age-specific fertility is not available in the earlier part of the period, but estimates are required over the whole period to make population projections. We therefore derive estimates of annual age-specific fertility rates using an indirect standardisation approach. Full details are given in Murphy (2016a, 2016b). We use fertility and mortality schedules for populations with both sexes combined rather than just for women as in most applications, but this makes no difference to our substantive conclusions. We calculated annual single year of age net migration rates using the balancing equation since population change and age-specific deaths are available.

### Projections

To assess the effects of choice of base year on population ageing up to the latest available year of around 2015, we undertook three population projection scenarios with base years of 1850 and every 10^th^ year between 1900 and 1950. We measure time, *t*, from the baseline year, and *a*^*x*^ (*t*) is any age structure index, such as mean or median age, dependency ratio or proportion of population aged 65 and over, at time *t* for each scenario in the set (*Ac, Bf, Mf, Ff*) defined in Table 1. Note that *a*^*Ac*^(0) = *a*^*Bf*^(0) = *a*^*Mf*^(0) = *a*^*Ff*^ (*0*).

Models *Mf* (“Mortality fixed”) and *Ff* (“Fertility fixed”) are the counterfactual assumptions conventionally made, for example, by Valaoras (1950), Notestein (1960), Hermalin (1966), Bengtsson and Scott (2010), and Lee and Zhou (2017), for measuring the relative contribution of fertility and mortality to population ageing. Model *Bf* (“Both fixed”) is presented for two main reasons. The first is that future population dynamics depend on both initial population and subsequent rates. For example, a population with an older initial age structure will be relatively insensitive to changes in fertility rates but will be sensitive to mortality changes, whereas the reverse is the case for an initial younger low mortality population. If they experienced identical vital rates, the relative importance attributed to fertility and mortality rates would differ in these two populations. Therefore the effect of initial population structure needs to be controlled for in order to identify the effect of subsequent fertility and mortality rates. The three models of Table 1 and the actual population have the same initial baseline structure, but different subsequent vital rates. Differences in population size and structure between any pair of alternative models at a future time point show the specific effect of the determinants that differ between the models (including any interactions with other components). The relative contributions of only fertility and only mortality may be quantified by comparison of values for models *Mf* and *Ff* with model *Bf*. This allows the sensitivity of population ageing to changing fertility and mortality rates to be assessed after removing the confounding influences of initial age structure.

The projection model *Bf* with fixed fertility and mortality rates in the absence of migration will eventually result in a stable population that does not age, with structure determined only by the initial rates and independent of the initial population structure (Coale 1972). Projection models with fixed fertility and mortality rates may be used to show how an observed population moves towards the stable population state. The link to a main alternative approach for assessing the contribution of fertility and mortality change to population ageing, by comparing a range of static stable population models (Coale 1956), is therefore apparent, but projection models also show how population ageing evolves during the transition between initial and final states. Since the model *Bf* with constant rates will eventually produce a stable population that is non-ageing, the change in population ageing between the initial and final model *Bf* values shows the contribution of initial age structure to subsequent population ageing since the two populations have identical fertility and mortality rates. A similar result for the lack of importance for the initial population structure also holds for models *Mf* and *Ff* by the weak ergodicity theorem (Arthur 1982), even though the population structure will not be constant.

### Explicandum of population ageing

**Fig. 1:**
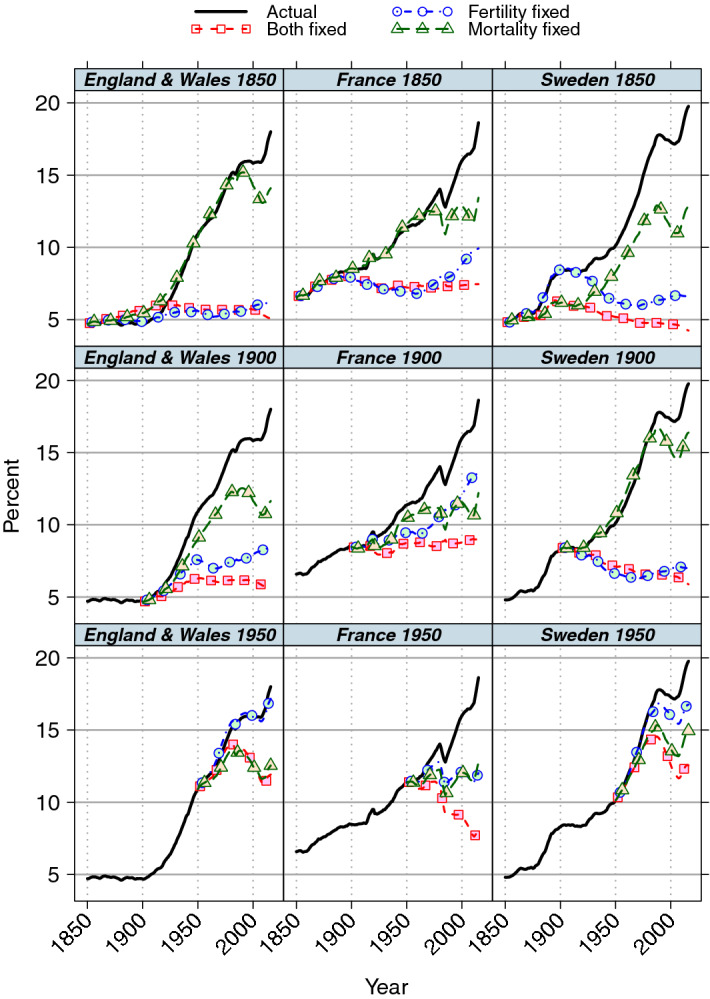
Proportion (%) aged 65 and over, observed and projected with rates fixed at baseline: base years 1850, 1900 and 1950

Most analyses using population projections have compared actual and predicted values with fixed fertility and actual mortality and vice versa. We illustrate this with projections for base years 1850, 1900 and 1950 in Fig. 1 for the three countries that we will discuss in more detail in the results section. These projections include estimates of net migration using annual age-specific net migration rates derived from using the balancing equation as discussed earlier (we also present projections with zero migration later). The trends for 1850 and 1900 bases are broadly similar in all countries; the fixed mortality model *Mf* is much closer to the observed value especially up to around 1980, apart from the France 1900 base where values are similar. Since the pace of population ageing appears to be largely unaltered if mortality is fixed rather than varying, the effect of subsequent mortality change on population ageing is interpreted as being minor. On the other hand, model *Ff* with fixed fertility but varying mortality shows large differences between observed and projected values indicating that population ageing is sensitive to fertility change over this period. These results are similar to other studies using counterfactual projections, which have concluded that fertility decline was the primary determinant of population ageing in developed societies over the twentieth century.

### Quantifying the relative contribution of fertility and mortality

Conclusions on the drivers of population ageing using counterfactual projections have usually been made by comparing projected and observed ageing indices at some future time with those at an earlier time point, usually the baseline value. As noted earlier, population ageing is determined by rates experienced after the base year and by the initial population structure. The constant rates projection model *Bf* depends only on initial structure and vital rate values, and subsequent migration rates. Models *Mf* and *Ff* differ from model *Bf* only by having variable fertility and mortality, respectively. Therefore, the differences between model *Bf* and models *Mf* and *Ff* reflect only differences in vital rates in the projection period. For example, the case of Sweden 1950 base (Fig. 1) shows that considerable population ageing would have occurred after 1950, even if fertility and mortality had remained constant because the initial age distribution was very different from that of the corresponding stable population. If this is not allowed for, the contribution of initial population structure will be incorrectly attributed to later fertility or mortality change. Population momentum effects are well-recognised in the case of population growth, but the similar effects on population ageing have received less attention, but see Rowland (1996).

The justification for the primacy of fertility change has been based on presentations such as Fig. 1, although the contributions of mortality and fertility cannot be quantified, which makes comparisons between different countries and time periods problematic. We measure these contributions as follows. The change in population ageing between the baseline time *t* = 0 and the final year *t* = *T* is the change in the observed age structure indicator over time, *a*^*Ac*^ (*T*) − *a*^*Ac*^ (*0*) (Table 1). This overall change, which includes the effect of the initial age structure and subsequent demographic rates on population ageing, may be decomposed as follows:

1. since the initial age structure and net migration rates are the same in both models *Ac* and *Bf*, the joint effect of the vital component rates of fertility and mortality, after the base year is
  1 $$ a^{Ac} \left( T \right) \, {-} \, a^{Bf} \left( T \right) $$
2. the joint effect of the other two determinants, initial age structure and net migration rates (although age structure has tended to be more influential than migration in practice), which we subsequently refer to as the non-vital component,
  2 $$ a^{Bf} \left( T \right) \, {-} \, a^{Ac} \left( 0 \right) $$

The change in population ageing due to mortality change since the base year may be calculated as the difference between scenarios with the same initial conditions, migration and fertility rates, but different mortality rates, actual (model *Ac*) and fixed (model *Mf*), i.e. *a*^*Ac*^ (*T*) − *a*^*Mf*^ (*T*). An alternative mortality measure may be calculated using fixed rather than actual fertility rates, i.e. *a*^*Ff*^ (*T*) − *a*^*Bf*^ (*T*), using models *Ff* and *Bf*. Both estimates compare the contribution of mortality fixed at baseline to that of actual mortality while having the same remaining variables.We use the average of these two estimates as the indicator of ageing due to mortality change

3 $$ 0.5\left( {a^{Ac} \left( T \right){-}a^{Mf} \left( T \right) + a^{Ff} \left( T \right){-}a^{Bf} \left( T \right)} \right) $$

The same analysis, with fertility and mortality reversed, gives the corresponding indicator of ageing due to fertility change

4 $$ 0.5\left( {a^{Ac} \left( T \right){-}a^{Ff} \left( T \right) + a^{Mf} \left( T \right){-}a^{Bf} \left( T \right)} \right) $$

While there are a number of alternative ways of decomposing demographic variables (Andreev et al. 2002; Caswell 1989; Caswell and Sánchez Gassen 2015; Horiuchi et al. 2008; Jdanov et al. 2017), the method above has the property that the sum of the fertility and mortality components (3) and (4) is equal to the total vital rate component (1) above; therefore it partitions the contributions of fertility and mortality to population ageing into two distinct components. It is also a simple index using only the four final values at time *T* and the initial value such as those shown in Fig. 1. Note that the same result holds for any intermediate time t* between the initial and final projection years.

Thus, the total change in population ageing between initial and final years may be separated into the sum of a vital rates component (1), which can be further disaggregated into the fertility and mortality terms, (3) and (4), and the residual non-vital component (2) that includes migration and initial age structure terms. The proportion of total change due to non-vital component and the proportions of the vital rates change component due to fertility and mortality may be calculated.

Analyses to date (Bengtsson and Scott 2010; Hermalin 1966; Lee and Zhou 2017; Notestein 1960; Valaoras 1950) investigating the role of fertility and mortality on population ageing have mainly compared the differences between the projected values of models *Ff* and *Mf* in Table 1 with baseline values, i.e. *a*^*Ff*^ (*T*) − *a*^*Ac*^ (*0*) and *a*^*Mf*^ (*T*) − *a*^*Ac*^ (*0*). As noted above, such comparisons do not isolate the contributions of fertility and mortality in the intervening period since initial population structure and net migration also contribute. However, this problem is mitigated if the initial age structure is similar to that of the constant rates stable population, i.e. *a*^*Bf*^ (*T*) is close to *a*^*Ac*^ (*0*), which is the first component of expressions (3) and (4). Most of the studies using counterfactual projections have used a baseline of 1900: although the reason for that particular choice is unspecified, it has the advantage of enabling generalised statements about the twentieth century to be made. However, the choice turns out to be fortunate since comparison of later results with the initial value and those of the more appropriate model *Bf* are very similar (Fig. 1), but it is clear that this would not be the case with a baseline such as 1950, potentially leading to problems in interpreting results of such studies.

### Treatment of migration

While migration influences population structure, the general conclusion is that such effects have been small with the levels of migration observed in practice (Goldstein 2009; Kisker 1950), although Swanson et al. (2016, p. 244) come to a different conclusion. The main interest has been in the relative contribution of fertility and mortality to population ageing: although migration can be important for overall population size and it has become of interest for future patterns (Murphy 2016a; United Nations Population Division 2000), but this issue is not the central concern of this paper.

Migration must be included to provide a comprehensive overview of the demographic determinants of population ageing. In addition, although the long-term impact of migration on population structure has been generally small in Europe, net migration rather than natural change is currently the dominant component of population change and it has a considerable impact on short-term indicators of ageing. In Sweden in 2015 the crude rates per 1000 population were 8.1 for net migration and 2.4 for natural increase (Eurostat 2018) and the proportion aged 65 and over increased by 1.4 per 1000, about half what it would have been with zero net migration. In that year, to have the same effect on population ageing as actual migration, an increase of over 60 per cent in fertility rates or reduction of over 20 per cent in mortality rates would have been required. In such circumstances, migration may complicate analysis and presentation of findings on long-term effects of fertility and mortality without contributing to their explanation. On the other hand, population ageing, as measured by the proportion of the population aged 65 and over, reversed in Sweden between 1988 and 2000 even though mortality continued to improve (Fig. 1). While this trend reversal was reinforced to some extent by positive net in-migration and a minor increase in fertility, the main reason was the reduction in births in the 1920s resulting in smaller birth cohorts reaching age 65 in that period.

Exclusion of migration allows the relative contribution of fertility and mortality to be identified more clearly. However, it is not as straightforward to incorporate migration into such analyses. There are alternative ways of operationalizing counterfactual migration scenarios. While counterfactual fertility and mortality analyses are almost always based on continuation of baseline values, the corresponding migration assumption is usually of zero net migration (this is, for example, one of the set of alternative projections produced by the United Nations Department of Economic and Social Affairs (2019) and by many other official agencies as well). Constant migration scenarios using baseline values could be operationalised using, for example, fixed numbers or fixed net migration rates. The results can be substantially different because the population sizes of the different counterfactual scenarios may be very different; for the 1900 base series, by 2016 the model *Ff* population size of Sweden is 15 times that of model *Mf*. The appropriate choice for a fixed non-zero migration scenario is therefore more arbitrary than in the case of fertility or mortality.

It is not clear how migration was treated in some of the earlier counterfactual projections studies. Some appear to apply just fixed fertility and mortality rates to an initial population, i.e. assume no net migration, and compare these results with later observed values that include the impact of migration in the intervening period, which means that the specific effects of fertility and mortality cannot be identified. The problems with specifying the appropriate way of incorporating migration are not unique to counterfactual population projection approaches. Standard stable population theory, as set out, for example, in Coale (1972) and Preston et al. (2001, Chapter 7), excludes migration. The lack of a preferred way of including migration within the stable population framework is reflected in the number of alternative specifications (Alho 2008; Bradatan 2016; Cerone 1987; Espenshade 1986; Espenshade et al. 1982; Sivamurthy 1982; Swanson et al. 2016).

The Preston, Himes and Eggers model includes migration in a consistent way based on net migration rates; therefore we also made projections including fixed net migration rates rather than absolute numbers. The values in Fig. 1 are based on comparisons with observed populations that include migration, as have since most analyses to date, but we also present results later for three countries, England and Wales, France and Sweden, based on synthetic zero-migration populations: (a) to estimate the effect of migration on age structure over extended time periods; and (b) to remove the confounding effect of migration in counterfactual projections, especially when analysing short periods. Zero migration populations are subject to observed levels of fertility and mortality, but net migration is assumed to be zero from the start dates of the series. Levels of international migration were low for at least a century before 1850, so the population distributions with and without migration are similar at 1850 (van Lottum 2007). These three countries exhibited different patterns of fertility, mortality and migration over the subsequent period, with France experiencing much earlier fertility decline, and Sweden much earlier mortality improvement. In the latter part of the nineteenth century Sweden had substantial out-migration, but overall net migration was close to zero in England and Wales and slightly positive in France (Murphy 2016a). These corresponding results with zero migration assumptions for the figures in the main text are shown in the Appendix, but they do not change the main conclusions derived below.

## Results

**Fig. 2:**
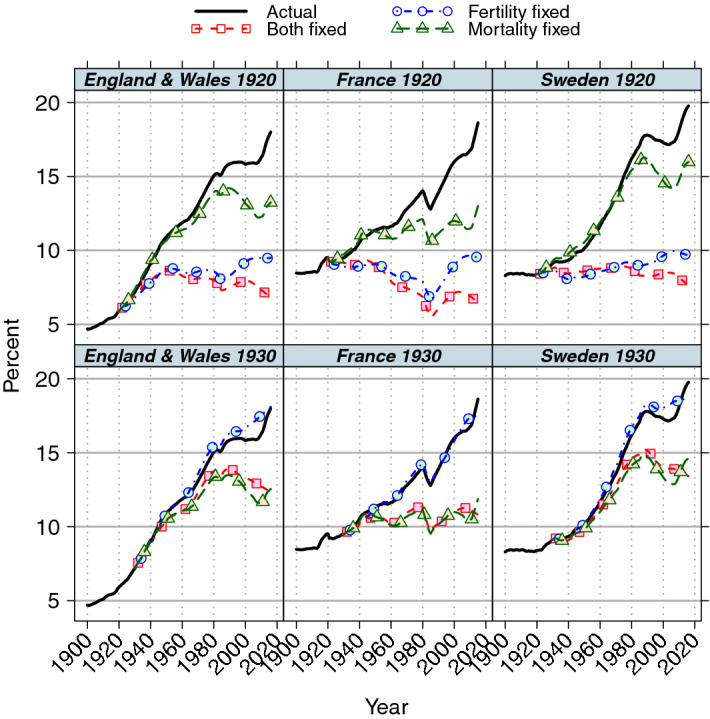
Proportion (%) aged 65 and over, observed and projected with rates fixed at baseline: base years 1920 and 1930

Fertility change was more important than mortality change for the analyses with bases starting at 1850 and 1900, but mortality change became more important with base starting at 1950 (Fig. 1). In particular, for England and Wales, mortality change appears to explain all change since 1950. More detailed analysis shows that the period when the importance of fertility and mortality reverses is between the 1920 and 1930 base years for England and Wales and Sweden, while the increasing importance of mortality change was more gradual and earlier in France (Fig. 2). The standard interpretation of results for the countries presented here would be that fertility change was primarily responsible for population ageing in the period 1920–2015, while mortality change was responsible in the overlapping period 1930–2015. Examination of results for the period 1920–30 does not resolve this apparent inconsistency since the period was short, population ageing was not substantial and analysis of 1920-based counterfactual projections over the 1920s suggests that fertility and mortality made similar contributions to population ageing in that decade. The conclusions about the determinants of population ageing over the past century or so are heavily dependent on an apparently arbitrary choice of base year. The main reason is the sharp drop in period fertility that occurred in that decade: for example, the TFR in Sweden fell by 1.3 children per woman, from 3.22 to 1.96, and by 0.5 and 0.4 of a child in England and Wales and France, respectively (INED 2018), while the change in mortality was much less marked and in line with long-term trends. In all countries, fertility levels around 1920 were higher than values at the end of the projection period, but levels around 1930 were slightly lower in England and Wales and Sweden, so the 1930 TFR values were close to the average of values in the period since 1930. The consequence is that projections with constant 1930 fertility values are more similar to the actual values in the period up to 2015, whereas 1920 fertility leads to a much younger population structure. Therefore, results using 1920 fertility appear to show a substantial fertility effect on population ageing over the past century or so, but 1930-based fertility analyses show limited effects. Although 1920 might be considered atypical given the recency of WWI and the 1919 influenza pandemic, this does not account for these results. Similar conclusions are found if, for example, 1910 rather than 1920 had been used for comparisons.

In the remainder of this paper, we extend the analysis to eight additional countries; consider formal and substantive implications of including or excluding migration; and how the contribution of fertility and mortality to population ageing may be better-quantified. We start by assessing the sensitivity of results to the choice of ageing indicator.

### Index of ageing

Figures 1 and 2 show one indicator of population ageing, the proportion aged 65 and over. There are substantial fluctuations in France around 1980, due to the small WWI birth cohorts crossing the age 65 boundary so their effect changes from increasing to retarding ageing (the annexation and subsequent return of Alsace-Lorraine in WWI also affected these trends). This age cut-off is arbitrary in that similar patterns would have been observed but at a different time period if an alternative indicator of ageing such as median age had been used. Other measures, such as old age dependency ratios (Hermalin 1966) and median or mean age, have been used but proportion aged 65 and over has been widely used to date. Mean age has advantages over alternative indicators (Murphy 2017) including that it gives more weight in calculations to those at the ends than to those in the middle of the age distribution, but may be less relevant if the main interest is in trends of older people. The Preston, Himes and Eggers model provides a decomposition only of population mean age so comparison with this method must use this index. Alternative results using mean age are shown in Figs. 4 and 5 in the Appendix. They show that similar qualitative conclusions hold about the relative importance of fertility and mortality if mean age had been used rather than the proportions aged 65 and over shown in Figs. 1 and 2.

### All-country analysis

Before presenting more detailed results for the three chosen countries, we show summary values with 1900 base year for all 11 countries with data starting before 1900 in HMD (Table 2). These results show that the projections with fixed mortality (*Mf*) are much closer to the observed values in the final year than the models that include fixed fertility (*Ff* and *Bf*), and therefore that fertility change was likely to be the main driver of population ageing over the period. We also show the proportions of vital change attributed to fertility and mortality using Eqs. (3) and (4). This confirms that the non-vital component (migration and initial population structure combined) had a generally small effect on population ageing, and that fertility change dominates, responsible for about three quarters of vital rate change on the proportion aged 65 and over on average. Table 5 in the Appendix shows that if mean age is used, the average proportion attributed to fertility is much higher, about 95 per cent.

**Table 2:** Components of population ageing (per cent aged 65 and over) due to non-vital component, fertility and mortality for 11 countries: projections from base year 1900

Country	Final Year	Actual per cent aged 65 and over:		Difference per cent aged 65 and over between final and initial years:				Non-vital component^a^ as per cent of Actual change	Per cent of Vital change due to:	
		Initial year (1900)	Final year	Actual (Ac)	Fertility and mortality fixed (Bf)	Fertility fixed (Ff)	Mortality fixed (Mf)		Fertility	Mortality
		(1)	(2)	(3)	(4)	(5)	(6)	(7)	(8)	(9)
Denmark	2016	6.7	18.8	12.1	− 1.7	− 0.6	8.4	− 13.8	82.5	17.5
England and Wales	2016	4.7	18.0	13.3	0.9	3.8	7.0	6.4	62.8	37.2
Finland	2015	5.3	19.9	14.7	0.4	0.8	12.0	2.4	89.1	10.9
France	2015	8.5	18.6	10.2	0.5	5.2	3.7	5.3	42.5	57.5
Iceland	2016	6.6	13.9	7.3	− 1.3	1.7	2.4	− 18.1	54.1	45.9
Italy	2014	6.3	21.4	15.1	− 1.5	− 0.5	9.2	− 10.1	79.0	21.0
Netherlands	2016	6.0	18.2	12.2	− 0.8	0.1	8.9	− 6.7	83.8	16.2
Norway	2014	7.8	15.9	8.1	− 2.8	− 2.1	5.6	− 35.0	85.2	14.8
Scotland	2016	4.9	18.5	13.6	0.7	3.2	7.8	5.0	67.6	32.4
Sweden	2016	8.3	19.8	11.5	− 2.4	− 1.3	8.1	− 21.0	83.9	16.1
Switzerland	2016	5.8	18.0	12.2	− 1.9	1.1	4.6	− 15.3	62.4	37.6

Column (3) = Column (2) - Column (1)

Column (7) = 100*Column (4)/Column (3)

Column (8) = 50*(Column (3) - Column (4) + Column (5) - Column (6))/(Column (3) - Column (4))

Column (9) = 50*(Column (3) - Column (4) - Column (5) + Column (6))/(Column (3) - Column (4))

^a^Includes migration (if any) and initial population structure; construction of all measures discussed in text

While the trends using proportions aged 65 and over and mean ages are generally similar, the proportion attributed to fertility rather than to mortality is much greater using population mean age. Reasons for this include the fact that proportion aged 65 and over is much more sensitive to patterns among older than younger people, which is why a large increase in births is required to make a noticeable change as in the case of Sweden in 2015 earlier. In addition, improvements at older ages occurred later than at younger ages, whereas the mean age depends on patterns at both ends of the age distribution.

The three countries chosen for more detailed analysis have high, medium and low values for proportion of population ageing attributed to fertility, and therefore reflect the range of experiences across Europe (excluding Eastern Europe) over this period. France is an outlier with mortality having more influence than fertility, whereas Sweden has amongst the lowest contribution of mortality in this period, reflecting their different demographic histories.

### Sensitivity of population size to base year and model assumptions

**Fig. 3:**
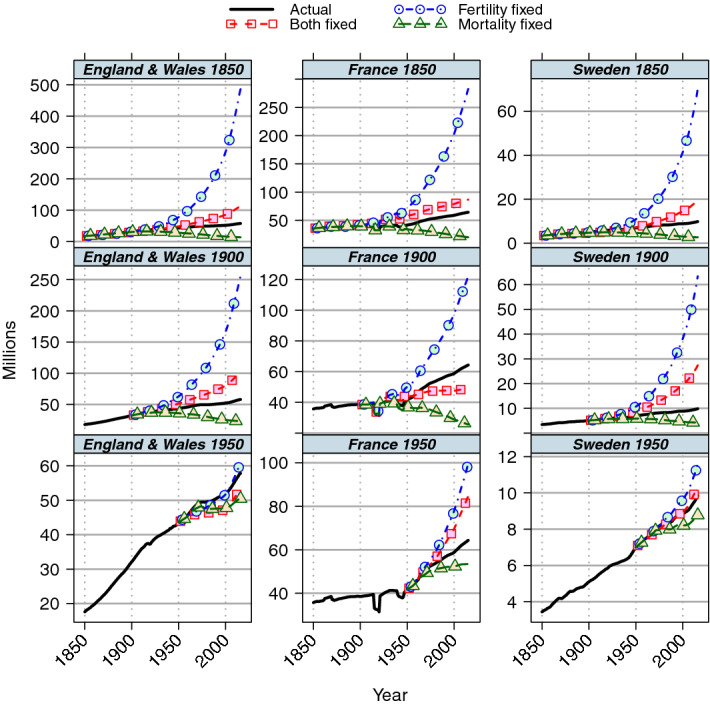
Population size of alternative projections, rates fixed at baseline: base years 1850, 1900 and 1950

**Fig. 4:**
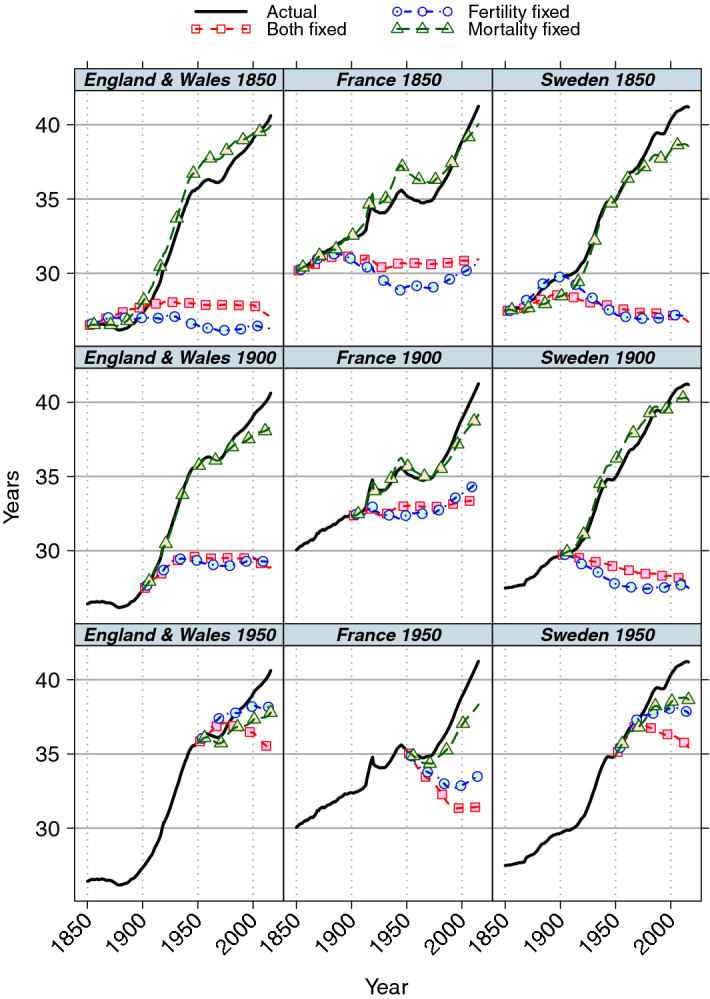
Mean age (years), observed and projected with rates fixed at baseline: base years 1850, 1900 and 1950 (c.f. Figure 1)

**Fig. 5:**
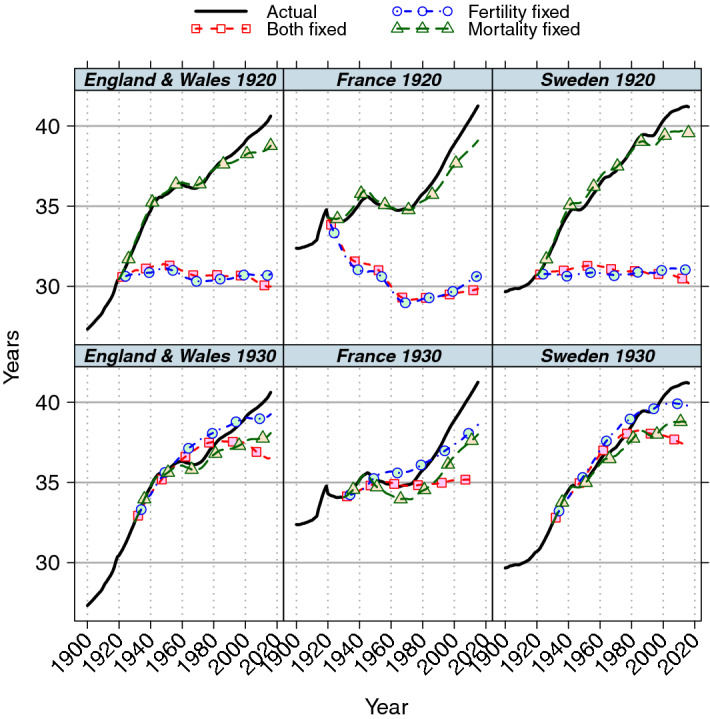
Mean age (years), observed and projected with rates fixed at baseline: no migration populations, base years 1920 and 1930 (c.f. Figure 2)

Figure 3 shows the sizes of the populations of models *Bf*, *Mf* and *Ff* together with the observed values for base years of 1850, 1900 and 1950 for the three selected countries. The final year projections for the model with mortality fixed at 1900 level for all countries and the model with fixed fertility and mortality for France are lower than the actual final values. However the projections with fixed fertility are considerably higher around 2015, 12 times the size of the 1900 population in Sweden, eight times in England and Wales, and three times in France. The high values appear to be implausible in the context of contemporary developed societies and therefore comparisons such as those of Fig. 1 are made with unrealistic population structures, suggesting such results should be interpreted cautiously as counterfactuals.

Extended timescales are necessary to mitigate the effect of initial population structure on the estimates of population ageing. However, conclusions using comparisons over long timescales will be with projected populations with sizes tending to zero or infinity and based on very different demographic parameters. If the effect of fertility change on population structure is assessed by a projection with fertility fixed at around 1850 levels, the projected populations for the countries considered here would be about eight times as large as the actual populations by 2015 and they would have very young age structures. On the other hand, if mortality is fixed at 1850 values then the projected populations would be about one quarter of the actual sizes.

However, with short projection periods, results can be very sensitive to confounding due to initial age structure and disturbances due to migration, although vital rates at the chosen base year are likely to be closer to those in the projection period. Counterfactual projections would therefore appear to be most useful in the intermediate period where the inherent problems with analyses over short or long projection periods will be reduced. We now consider how some of these limitations may be reduced.

### Influence time and analysis time

If we are interested in identifying the determinants of population ageing between two time points *t*_*1*_ and *t*_*2*_ (analysis time), a decision is required about the period over which the determinants in this period should be estimated (influence time). Population ageing is the shifting of the population age distribution to older ages, which is a continuous process that may be measured by, for example, the first derivative of the population mean age, so the analysis interval (*t*_*1*_*, t*_*2*_) may be arbitrarily small. Clearly the pace of population ageing does not just depend on the current instantaneous rates of fertility or mortality, but also on the full lifetime experiences of those alive, therefore it is directly influenced by fertility and mortality experience for a period up to around 100 years earlier. The rates in a given year have a weak formal relationship with the age distribution in that year so their influence on population ageing in that year may be small. Therefore very short-term projections can be inappropriate for identifying the determinants of population ageing. Not recognising the distinction between influence and analysis times risk confounding the effects of demographic rates and initial population structure, making it difficult to identify the specific role of these rates on population ageing. However, presentation of results based on extended projection intervals of a century or so does not provide information on time trends, which may have changed substantially over such time scales.

Lee and Zhou (2017) use a short 5-year interval, 2005 to 2010, for analysis time, but use projections with base year of 1900, the start of the influence time period, to assess the wider role of fertility and mortality rates on population ageing. Fertility and mortality rates for the previous 100 years were used for comparison with the analyses of Preston and Stokes (2012) who used the same 5-year window. However, Lee and Zhou (2017) identify more general advantages for such a choice. For example, if two countries had different initial age structures but identical vital rates subsequently, estimates of the determinants of population ageing based on a period just after the base year would differ, but those using a window around the end of the projection period would be similar since both population structures will tend to converge. The time for achievement of stability—relaxation time—varies between demographic indicators. In particular, population aged 65 and over is strongly determined by events more than 65 years ago and will therefore take especially long lead times to become a fixed proportion of the total population.

Results using short analysis times can provide estimates for short intervals such as single years, whereas the standard approach using projections over extended periods from the base year just produces an average over the whole period. However, a disadvantage of concentrating on such relatively short analysis time windows is that an influence interval of around one hundred years of prior data is required to allow for the full direct impact of earlier rates before the first analysis time estimate can be calculated (as is also the case for the Preston, Himes and Eggers model). This is one reason why the main analyses here are confined to countries with over 150 years of available data. Another problem is that large short-term migration fluctuations in the analysis interval can mask the effects of earlier fertility and mortality trends as demonstrated by the case of Sweden earlier. We will consider some empirical evidence on these issues.

To investigate the implications of different analysis times, we use the six bases from 1900 to 1950 for the usual three models. We present averages of the components of population ageing by treating each year from 1960 up to the last available year as the final projection year, with influence times spanning one and 5 years before the final projection year and the base year (Table 3). Because of the clear difference in findings with bases in the first and second quarters of the twentieth century, we present these groups separately. The 1- and 5-year windows provide very similar results on the proportion of change attributed to fertility and mortality as expected, and the substantial reversal of the relative influences of fertility and mortality between the two base periods is apparent for England and Wales and Sweden, with a smaller change for France. However, the analyses from base to final year are less interpretable, especially for later base years when all the countries show that the effect of fertility change was to retard rather than to promote population ageing. While the magnitude was small in France and Sweden, in England and Wales, the estimated effect of fertility change on population ageing was substantially negative. While there was some minor increase in fertility, it should be emphasised that non-vital effects, especially initial structure, dominated; and in addition these results are based on projection horizons as short as 10 years in some cases.

**Table 3:** Components of population ageing (per cent aged 65 and over) due to due to non-vital component, fertility and mortality: projections from base years 1900–25 and 1925–50 to final years 1960–2015/16

Country	Bases	Span	Average difference^a^ per cent aged 65 and over between final and initial years:				Non-vital component^b^ as per cent of Actual change	Per cent of Vital change due to:	
			Actual (Ac)	Fertility and mortality fixed (Bf)	Fertility fixed (Ff)	Mortality fixed (Mf)		Fertility	Mortality
			(1)	(2)	(3)	(4)	(5)	(6)	(7)
England and Wales	Pre−1925	1 year	0.1	0.0	0.0	0.0	− 16.5	60.2	39.8
		5 years	0.7	− 0.1	0.1	0.3	− 16.1	62.9	37.1
		Base year	9.9	2.0	3.5	7.0	20.0	71.6	28.4
	Post−1925	1 year	0.1	0.0	0.1	0.0	22.9	5.4	94.6
		5 years	0.7	0.2	0.7	0.2	29.4	4.8	95.2
		Base year	6.1	4.2	7.2	3.3	69.3	− 55.8	155.8
France	Pre−1925	1 year	0.2	0.0	0.1	0.1	5.2	52.8	47.2
		5 years	0.8	0.0	0.3	0.4	− 4.5	54.6	45.4
		Base year	6.0	− 0.5	2.2	2.3	− 8.3	51.0	49.0
	Post−1925	1 year	0.2	0.0	0.1	0.1	5.5	39.2	60.8
		5 years	0.8	0.1	0.6	0.3	13.2	31.6	68.4
		Base year	4.4	0.8	4.6	0.7	19.2	− 5.9	105.9
Sweden	Pre−1925	1 year	0.1	0.0	0.0	0.1	− 22.5	72.3	27.7
		5 years	0.8	− 0.1	0.0	0.5	− 14.2	73.9	26.1
		Base year	8.0	− 1.0	− 0.4	6.4	− 12.4	87.7	12.3
	Post−1925	1 year	0.1	0.0	0.1	0.1	14.9	38.5	61.5
		5 years	0.8	0.3	0.6	0.4	30.5	33.6	66.4
		Base year	6.8	4.1	7.1	3.9	60.8	− 8.8	108.8

^a^Difference is between values for final year and previous Span years before final year or Base year as appropriate

^b^For definitions, see Table 2

Although population ageing occurred between the set of base years 1930–50 and the final years of 1960–2015/6 in Table 3, this was substantially accounted for by the rates before the base year that determined the initial population structure rather than rates after the base year. This is reflected in the mismatch between age distributions of the actual population at baseline and of the stable population based on observed vital rates at baseline. If they were similar, the trend of the fixed fertility and mortality scenario model, *Bf*, would be horizontal, i.e. no population ageing, as seen, for example, in the 1850-based projections (Fig. 1). Population dynamics with later base years—and therefore short- or medium-term estimates—are influenced by the relaxation of the younger observed age distribution towards the older stable population structure, as well as fertility or mortality change in the intervening period, see Figs. 1 and 2, and Table 2. Therefore, estimates of population ageing from projections even with influence and analysis times of several decades give little useful information about the role of vital rates within the projection window without further adjustment. The corresponding results using mean age (Table 6 in the Appendix) are broadly similar, with 1- and 5-year values showing generally consistent results between countries and across time, although the proportion of population ageing attributed to fertility is generally higher than with the proportion aged 65 and over indicator, as observed in Table 2.

**Fig. 6:**
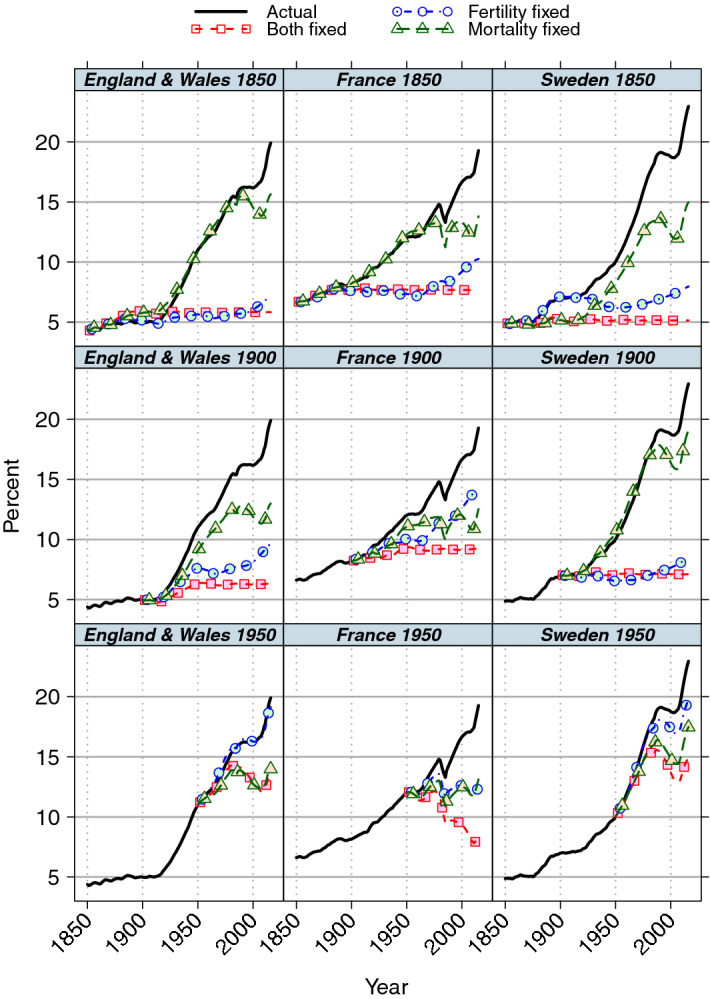
Proportion (%) aged 65 and over, observed and projected with rates fixed at baseline: Zero migration population, base years 1850, 1900 and 1950 (c.f. Figure 1)

Finally, as above we compare results with and without migration, and with alternative indicators of ageing by averaging projections from the 1900 to 1950 bases for the three countries over all years from 1960 to the final date (Table 4). These results again confirm that the estimated fraction of population ageing attributed to fertility rather than mortality is considerably higher when using the indicator of mean age than proportion aged 65 and over. However, both indicators show that the magnitude and pace of population ageing is currently lower than if there had been no migration. Comparison of Fig. 1 and Fig. 6 in the Appendix shows that the level of population ageing is particularly sensitive to migration trends in Sweden. Heavy out-migration in the later nineteenth century led to lower numbers of people under age 65 in the population in the early twentieth century and therefore increased the rate of population ageing. More recently, positive in-migration of mainly young people has made the population structure younger so decreasing the rate of population ageing it in the current century. Migration also had a substantial impact on population size (Fig. 3 and Fig. 8 in the Appendix) in Sweden, with recent in-migration only partially compensating for the earlier out-migration.

**Table 4:** Components of population ageing, mean age (years) and per cent aged 65 and over, by migration status: projections from base years 1900–50 to final years 1960–2015/16

Country	Index	Migration type	Difference between final and initial years				Non-vital component as per cent of actual change	Per cent of Vital change due to:	
			Actual (Ac)	Fertility and mortality fixed (Bf)	Fertility fixed (Ff)	Mortality fixed (Mf)		Fertility	Mortality
England and Wales	Mean age	With migration	13.3	1.6	2.0	11.1	11.7	88.7	11.3
		No migration	14.4	2.1	2.8	12.0	14.7	87.3	12.7
	Proportion aged 65 and over	With migration	13.3	0.9	3.8	7.0	6.4	62.8	37.2
		No migration	14.9	1.3	4.6	8.0	9.0	62.4	37.6
France	Mean age	With migration	8.9	1.1	2.4	6.8	12.4	78.4	21.6
		No migration	9.1	1.1	2.5	6.8	12.1	76.9	23.1
	Proportion aged 65 and over	With migration	10.2	0.5	5.2	3.7	5.3	42.5	57.5
		No migration	11.1	1.1	6.0	4.4	9.8	42.1	57.9
Sweden	Mean age	With migration	11.5	− 1.9	− 2.2	10.5	− 16.7	97.1	2.9
		No migration	14.6	0.2	0.1	13.3	1.5	95.8	4.2
	Proportion aged 65 and over	With migration	11.5	− 2.4	− 1.3	8.1	− 21.0	83.9	16.1
		No migration	16.0	0.1	1.5	12.1	0.8	83.5	16.5

For definitions, see Table 2

**Fig. 7:**
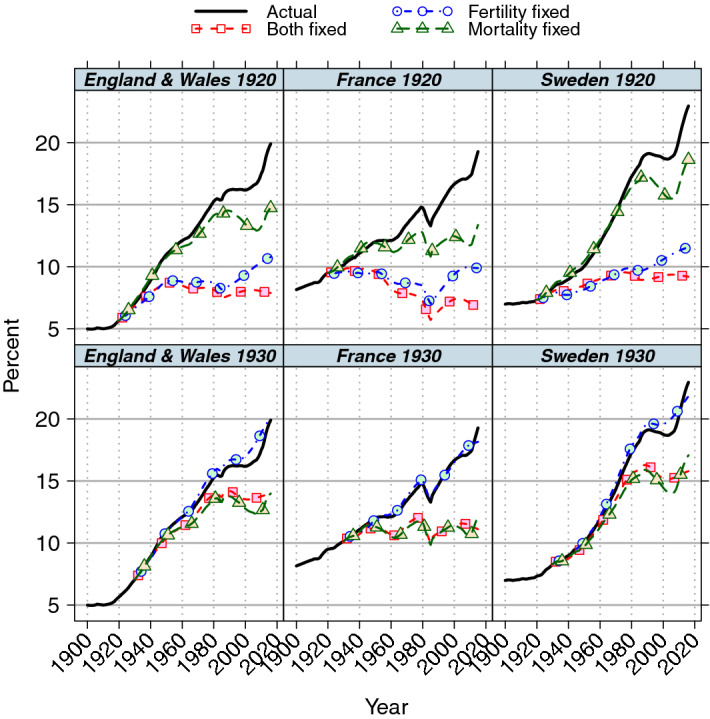
Proportion (%) aged 65 and over, observed and projected with rates fixed at baseline: Zero migration population, base years 1920 and 1930 (c.f. Figure 2)

**Fig. 8:**
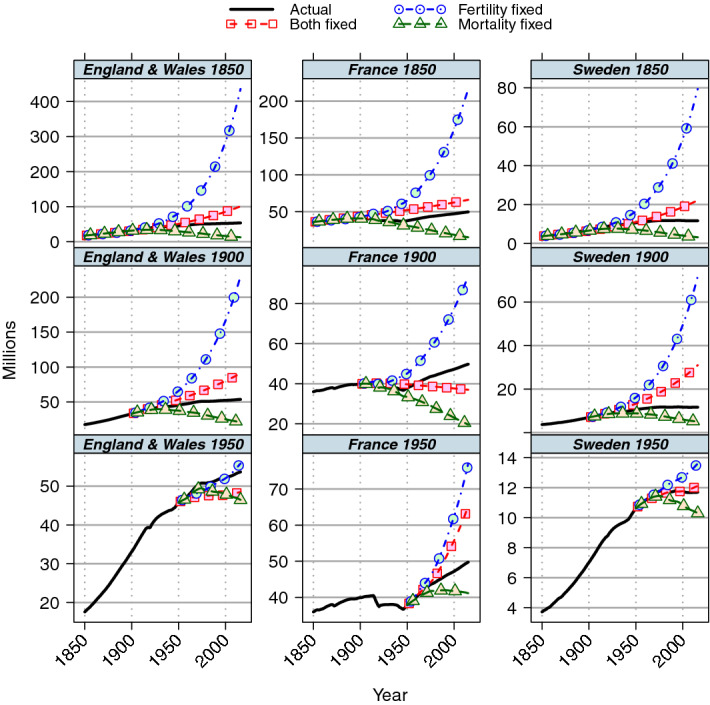
Population size of alternative projections, rates fixed at baseline: Zero migration population, base years 1850, 1900 and 1950 (c.f. Figure 3)

However, long-term ageing trends rather than levels are determined more substantially by fertility and mortality rather than migration in these countries. In all cases, results for analyses with and without migration show almost identical contributions of fertility and mortality over the base to final year period, including the marked difference between projections in the first and second quarters of the twentieth century (Fig. 2. and Fig. 7 in the Appendix). This was even in the case of Sweden where migration had a much greater effect than in the other countries, confirming the conclusion that typical patterns of migration have had little long-term effect on the other components of population ageing, even though the short-term effects can be substantial; compare Table 3 and Table 7 in the Appendix and for projections with equal influence and analysis periods and with 1-year analysis periods.

## Conclusions and Summary

Counterfactual population projections have been identified as the preferred method for establishing the primacy of fertility or mortality change as the driver of population ageing not only in high-income countries over the twentieth century, but also more generally (Population and Development Review 2017). Approaches using counterfactual population projections have attractive properties. The technology is readily available, widely understood and straightforward to present. The approach appears to simplify the analysis of the determinants of population ageing; for example, setting migration to zero means that the contributions of both the migrants and any descendants are removed without further adjustment (as long as this exclusion does not alter subsequent national fertility and mortality rates). However, counterfactual population projections have a number of substantive and formal limitations. For example, the relative importance of fertility and mortality to population ageing in recent decades in England and Wales and Sweden seems to depend substantially on whether we undertake the same analysis over the past 95 or past 85 years. The reason for the apparently strong fertility effect over the twentieth century arises because in the period before 1920, TFR was considerably higher than average subsequent values. In addition, the period fertility measures conventionally used as inputs to such projection models are highly sensitive to timing (“tempo”) changes, so are a poor basis for making long-term forecasts, which require consideration of quantum effects (Sobotka and Lutz 2011).

Analyses based on counterfactual population projection for a set of high-income countries with different base and final years, show that results are often inconsistent, sometimes considerably so, and we conclude that caution is needed before generalising from individual cases:

1. The results are highly sensitive to the population distributions and vital rates in the base year. A consequence is that apparently minor changes in choice of base year can lead to completely reversed conclusions about the determinants of population ageing.
2. The base year level of fertility, which is a period indicator, appears to be the main factor that determines whether the counterfactual projection approach indicates that fertility or mortality is the main driver of population ageing.
3. The projections approach does not appear to have a natural transitive property. The effects of fertility and mortality measured in sub-intervals do not add up to impact over the whole interval. This is in contrast to the Preston, Himes and Eggers model (Murphy 2017).
4. There has been no clear index for attributing changes to fertility or mortality (one possible index is derived and presented here). However, there is a strong case that comparisons should be of the actual value with those from a model with fixed fertility and mortality at the final time period, rather than with the actual baseline value as usually done.
5. The effect of migration on population structure over extended periods is small, confirming earlier empirical studies.
6. Different ageing indices tend to produce similar qualitative conclusions, but quantitative results may differ markedly.
7. The usual approach with identical influence and analysis times does not distinguish the effects of initial age structure and subsequent fertility and mortality rates. While this has not been a major issue for projections based around 1900, it is for those made some decades later.
8. Use of a short analysis window at the end of a long influence period mitigates some, but not all, of these problems, but this requires comprehensive detailed data for up to a century before definitive analyses can start to be made.

Counterfactual projections and stable population modelling have been used to argue for the primacy of fertility change on population ageing across high-income countries over the past century or so. However, some of the early work in this area was more nuanced. Kisker (1950, p. 57) concluded: “As for the relative importance of these factors [fertility, mortality and migration], much depends on the population and period of time under consideration”, and Thompson (1948) in the middle of the twentieth century stated although declines in US fertility were historically the most important, especially in the period 1920–50, in future declines in mortality may become the most important. This would appear to have been an accurate prediction and current trends suggest that mortality will continue to remain the primary driver of population ageing in high-income countries in the twenty-first century, and it is becoming increasingly important in middle-income countries as the historical legacy of high fertility on current population structure diminishes, while future fertility trends will remain crucial in the Least Developed Regions.

## Data Availability

The article is based on publicly available data, derived from Human Mortality Database (http://www.mortality.org/cgi-bin/hmd/DataAvailability.php).

## Appendix

**Table 5:** Components of population ageing, mean age (years), due to non-vital component, fertility and mortality for 11 countries: projections from base year 1900

Country	Final Year	Actual mean values:		Difference between initial and final years:				Non-vital component as per cent of Actual change	Per cent of Vital change due to:	
		Initial year (1900)	Final year	Actual (Ac)	Fertility and mortality fixed (Bf)	Fertility fixed (Ff)	Mortality fixed (Mf)		Fertility	Mortality
		(1)	(2)	(3)	(4)	(5)	(6)	(7)	(8)	(9)
Denmark	2016	28.2	41.3	13.1	− 1.5	− 1.8	12.0	− 11.6	97.3	2.7
England and Wales	2016	27.3	40.6	13.3	1.6	2.0	11.1	11.7	88.7	11.3
Finland	2015	27.4	42.3	14.9	0.3	− 1.5	15.3	2.2	108.0	− 8.0
France	2015	32.4	41.2	8.9	1.1	2.4	6.8	12.4	78.4	21.6
Iceland	2016	28.1	37.9	9.8	− 0.5	0.6	7.8	− 4.8	85.1	14.9
Italy	2014	28.4	44.4	16.0	− 1.3	− 2.8	14.9	− 8.0	101.2	− 1.2
Netherlands	2016	27.6	41.6	14.1	− 0.4	− 1.3	13.7	− 2.7	101.9	− 1.9
Norway	2014	28.2	39.6	11.4	− 2.1	− 2.2	10.6	− 18.0	97.5	2.5
Scotland	2016	27.0	41.8	14.8	1.3	1.8	12.7	9.1	90.3	9.7
Sweden	2016	29.7	41.2	11.5	− 1.9	− 2.2	10.5	− 16.7	97.1	2.9
Switzerland	2016	28.6	42.1	13.4	− 1.0	− 0.3	10.5	− 7.1	87.3	12.7

For definitions, see Tables 1 and 2

**Table 6:** Components of population ageing, mean age (years), due to due to non-vital component, fertility and mortality: projections from base years 1900–25 and 1925–50 to final years 1960–2015/16

Country	Bases	Span	Difference between initial and final years (years)				Non-vital component as per cent of Actual change	Per cent of Vital change due to:	
			Actual (Ac)	Fertility and mortality fixed (Bf)	Fertility fixed (Ff)	Mortality fixed (Mf)		Fertility	Mortality
			(1)	(2)	(3)	(4)	(5)	(6)	(7)
England and Wales	Pre−1925	1 year	0.1	0.0	0.0	0.0	− 3.3	64.3	35.7
		5 years	0.4	− 0.1	0.0	0.2	− 20.4	69.7	30.3
		Base year	9.5	1.7	1.8	8.7	18.3	94.8	5.2
	Post−1925	1 year	0.1	0.0	0.1	0.0	21.1	28.3	71.7
		5 years	0.4	0.0	0.3	0.2	10.9	34.4	65.6
		Base year	3.9	3.2	4.6	2.5	81.5	− 95.4	195.4
France	Pre−1925	1 year	0.1	0.0	0.0	0.1	1.4	64.0	36.0
		5 years	0.5	0.0	0.2	0.3	0.0	64.8	35.2
		Base year	4.5	− 0.9	− 0.4	3.4	− 21.2	84.2	15.8
	Post−1925	1 year	0.1	0.0	0.0	0.1	− 3.2	56.0	44.0
		5 years	0.5	0.0	0.2	0.3	− 3.4	56.0	44.0
		Base year	2.8	0.3	2.1	1.0	10.5	26.8	73.2
Sweden	Pre−1925	1 year	0.1	0.0	0.0	0.0	− 54.2	72.6	27.4
		5 years	0.4	− 0.1	0.0	0.3	− 29.5	76.5	23.5
		Base year	9.1	− 0.6	− 0.8	8.7	− 6.8	99.4	0.6
	Post−1925	1 year	0.1	0.0	0.0	0.0	− 18.9	47.1	52.9
		5 years	0.4	0.0	0.2	0.2	6.0	50.9	49.1
		Base year	5.2	3.5	5.0	3.7	68.5	10.4	89.6

For definitions, see Tables 1, 2 and 3

**Table 7:** Components of population ageing, mean age (years) and per cent aged 65 and over, by migration status: 1-year projection window from base years 1900–50 to final years 1960–2015/16

Migration status: Ageing index: Country	Difference between final and previous years:				Non-vital component as per cent of Actual change	Per cent of vital change due to:	
	Actual (Ac)	Fertility and mortality fixed (Bf)	Fertility fixed (Ff)	Mortality fixed (Mf)		Fertility	Mortality
1. With migration: mean age (years)							
England and Wales	0.17	0.02	0.05	0.11	15.19	69.41	30.59
France	0.16	0.02	0.07	0.12	14.15	66.81	33.19
Sweden	− 0.04	− 0.09	− 0.07	− 0.08	4.43	36.60	63.40
2. With migration: proportion (%) aged 65 and over							
England and Wales	0.23	0.00	0.05	0.11	23.12	62.16	37.84
France	0.44	0.01	0.07	0.39	43.89	86.14	13.86
Sweden	0.14	− 0.05	− 0.02	0.07	19.33	74.24	25.76
3. Zero migration: mean age (years)							
England and Wales	0.17	0.00	0.04	0.10	16.60	66.06	33.94
France	0.15	0.00	0.05	0.10	14.74	66.97	33.03
Sweden	0.13	0.00	0.03	0.07	12.90	64.10	35.90
4. Zero migration: proportion (%) aged 65 and over							
England and Wales	0.28	0.00	0.07	0.13	27.58	60.51	39.49
France	0.45	0.00	0.07	0.40	44.35	88.27	11.73
Sweden	0.32	0.00	0.05	0.21	31.85	75.30	24.70

For definitions, see Tables 1, 2 and 3 and text for definition of 1-year analysis interval
